# Saving Lives through Global Safe Water

**DOI:** 10.3201/eid1110.051099

**Published:** 2005-10

**Authors:** James M. Hughes, Jeffrey P. Koplan

**Affiliations:** * Emory University, Atlanta, Georgia, USA

**Keywords:** safe water, unsafe water, waterborne disease, sanitation, hygiene, diarrhea, pneumonia, millennium development goals, commentary

Unsafe water is a global public health threat, placing persons at risk for a host of diarrheal and other diseases as well as chemical intoxication. Unsanitary water has particularly devastating effects on young children in the developing world. Each year, >2 million persons, mostly children <5 years of age, die of diarrheal disease (*1**,**2*). For children in this age group, diarrheal disease accounted for 17% of all deaths from 2000 to 2003 (*3*), ranking third among causes of death, after neonatal causes and acute respiratory infections. Severe, prolonged diarrheal disease can also lead to malnutrition and impaired physical and cognitive development (*4*). Nearly 90% of diarrhea-related deaths have been attributed to unsafe or inadequate water supplies and sanitation (*5*)—conditions affecting a large part of the world's population. An estimated 1.1 billion persons (one sixth of the world's population) lack access to clean water and 2.6 billion to adequate sanitation (*5*).

At the 2000 United Nations (UN) Millennium Summit, member states adopted a set of 8 goals and related targets and indicators aimed at helping end human poverty and its ramifications (*6*). Among these Millennium Development Goals is a call to halve by the year 2015 the proportion of persons without sustainable access to safe drinking water and basic sanitation. Although some progress has been made, much remains to be done. Toward this end, in March 2005, the UN launched the "International Decade for Action: Water for Life 2005–2015." A UN Millennium Project Task Force has identified 5 guiding principles and 10 actions needed to intensify efforts to meet the targets (*7**,**8*). Building on lessons learned from the previous International Drinking Water and Sanitation Decade during the 1980s will also be an important part of this process. Success in reaching these targets will help achieve the other 7 Millennium Development Goals, increase workforce productivity, and substantially reduce the amount of time that women and children spend collecting and storing water, which will free them to pursue other productive and educational activities. Moreover, reaching these goals will be an important step toward breaking the cycle of poverty and disease.

A collaborative, interdisciplinary effort to ensure global access to safe water, basic sanitation, and improved hygiene is the foundation for ending this cycle. In addition to mobilizing political will among national leaders and heads of international agencies, this effort will require sustained involvement and commitment from a broad range of public and private-sector organizations, such as CARE and Procter & Gamble, which have long been involved in efforts to provide safe water in developing countries. Other corporations joining these efforts include The Coca-Cola Company and Starbucks, the latter through its recent purchase of Ethos Water, whose profits have supported safe water projects in India and several African countries. Identifying the specific roles and responsibilities of the many organizations and agencies with missions to improve access to safe water and sanitation will be critical to the success of this effort. The World Health Organization–sponsored International Network for the Promotion of Safe Household Water Treatment and Storage, a global collaboration of UN and bilateral agencies, nongovernmental organizations, research institutions, and the private sector (*8*), could serve as a model for improving coordination of international efforts in this area.

Innovative approaches toward improving water, sanitation, and hygiene must be implemented and evaluated. A number of studies conducted in a variety of geographic settings have shown that interventions such as point-of-use disinfection of water and educational efforts to improve personal hygiene help reduce disease prevalence (*9*). These studies also highlight the importance of tailoring such interventions to local situations. For example, a recent study in an area in rural western Kenya that had turbid source water found that household use of a flocculant-disinfectant preparation helped reduce the prevalence of diarrhea in children <2 years of age (*10*). Studies in refugee camps in Africa (*11*) and urban slums in Asia (*12*) have documented that handwashing with soap reduced the prevalence of diarrhea in all age groups (*11*) and lowered the incidence of diarrhea and pneumonia in children <5 years of age (*12*). The reduced incidence of pneumonia found in the second study is noteworthy and warrants further study. Although interventions for improving sanitation have lagged behind those for water, promising advances have been made, especially in the development of ecologic sanitation systems. Recent experiences with the tsunami in Asia and Hurricane Katrina on the US Gulf Coast are grim reminders of the need to address water and sanitation urgently following natural disasters.

The United States currently ranks last among the 22 member countries of the Development Assistance Committee of the Organization for Economic Cooperation and Development in net official development assistance provided to developing countries, when such assistance is measured as a percentage of gross national income (*13*). Today's political and social climate presents an important opportunity to improve this situation. As Barry Bloom, Dean of the Harvard School of Public Health, has written, "The United States should be investing efforts and funds to strengthen the health structures in countries around the world. This investment would protect our country and every other against global epidemics, save million of lives, and change the US image from one of self interest to one of human interest" (*14*).
